# Current therapy of KRAS-mutant lung cancer

**DOI:** 10.1007/s10555-020-09903-9

**Published:** 2020-06-16

**Authors:** Aron Ghimessy, Peter Radeczky, Viktoria Laszlo, Balazs Hegedus, Ferenc Renyi-Vamos, Janos Fillinger, Walter Klepetko, Christian Lang, Balazs Dome, Zsolt Megyesfalvi

**Affiliations:** 1 Department of Thoracic Surgery, National Institute of Oncology-Semmelweis University, Rath Gyorgy u. 7-9, Budapest, 1122 Hungary; 2 National Koranyi Institute of Pulmonology, Koranyi Frigyes u. 1, Budapest, Hungary; 3 Division of Thoracic Surgery, Department of Surgery, Comprehensive Cancer Center Vienna, Medical University Vienna, Waehringer Guertel 18-20, A-1090 Vienna, Austria; 4 Department of Thoracic Surgery, Ruhrlandklinik, University Clinic Essen, Essen, Germany

**Keywords:** KRAS mutation, Lung cancer, Targeted therapy, Predictive factor, Prognostic factor

## Abstract

KRAS mutations are the most frequent gain-of-function alterations in patients with lung adenocarcinoma (LADC) in the Western world. Although they have been identified decades ago, prior efforts to target KRAS signaling with single-agent therapeutic approaches such as farnesyl transferase inhibitors, prenylation inhibition, impairment of KRAS downstream signaling, and synthetic lethality screens have been unsuccessful. Moreover, the role of KRAS oncogene in LADC is still not fully understood, and its prognostic and predictive impact with regards to the standard of care therapy remains controversial. Of note, KRAS-related studies that included general non-small cell lung cancer (NSCLC) population instead of LADC patients should be very carefully evaluated. Recently, however, comprehensive genomic profiling and wide-spectrum analysis of other co-occurring genetic alterations have identified unique therapeutic vulnerabilities. Novel targeted agents such as the covalent KRAS G12C inhibitors or the recently proposed combinatory approaches are some examples which may allow a tailored treatment for LADC patients harboring KRAS mutations. This review summarizes the current knowledge about the therapeutic approaches of KRAS-mutated LADC and provides an update on the most recent advances in KRAS-targeted anti-cancer strategies, with a focus on potential clinical implications.

## Introduction

Over the past 20 years, the formerly prevalent and widespread pessimism regarding the therapeutic approaches and prognosis of advanced-stage non-small lung cancer (NSCLC) has changed dramatically with the development of molecular profiling, targeted therapeutic agents, immune checkpoint inhibitors, and precision medicine [1]. These efforts have offered valuable insights into the mutational landscape of NSCLC, including the Kirsten rat sarcoma viral oncogene homolog (KRAS) mutation, which is the most common gain-of-function alteration, accounting for approximately 30% of lung adenocarcinomas (LADCs) in Western countries and about 10% of Asian LADCs [2, 3].

KRAS protein, encoded by the KRAS proto-oncogene, is a small guanine triphosphatase (GTPase) that serves as a binary switch in signal transduction for most receptor tyrosine kinases including EGFR, MET, or ALK, and plays a key role in regulating various cell functions [4, 5]. Oncogenic mutations of the KRAS gene mostly occur in exon 2 at codon 12, less frequently at codon 13 (3–5%) and rarely at exon 3 codon 61 (less than 1%) [5]. These alterations are missense mutations that impair the ability of KRAS to hydrolyze GTP, resulting in the constitutive activation of its effector pathways and thus cancer development and progression [6]. Because of its high frequency in LADC, several preclinical and clinical investigations have been conducted, seeking effective therapeutic approaches targeting KRAS mutation. Still, to date, no effective RAS inhibitors are currently used in routine clinical practice and the approaches for treating KRAS-mutant LADC mirror those for treating NSCLC that lacks a known driver mutation. In this review, we systematically analyze the clinically relevant aspects of KRAS-mutant NSCLC, mainly focusing on the clinicopathological relevance, therapeutic implications, and new treatment opportunities.

## Clinical relevance of KRAS mutations in NSCLC

**Fig. 1:**
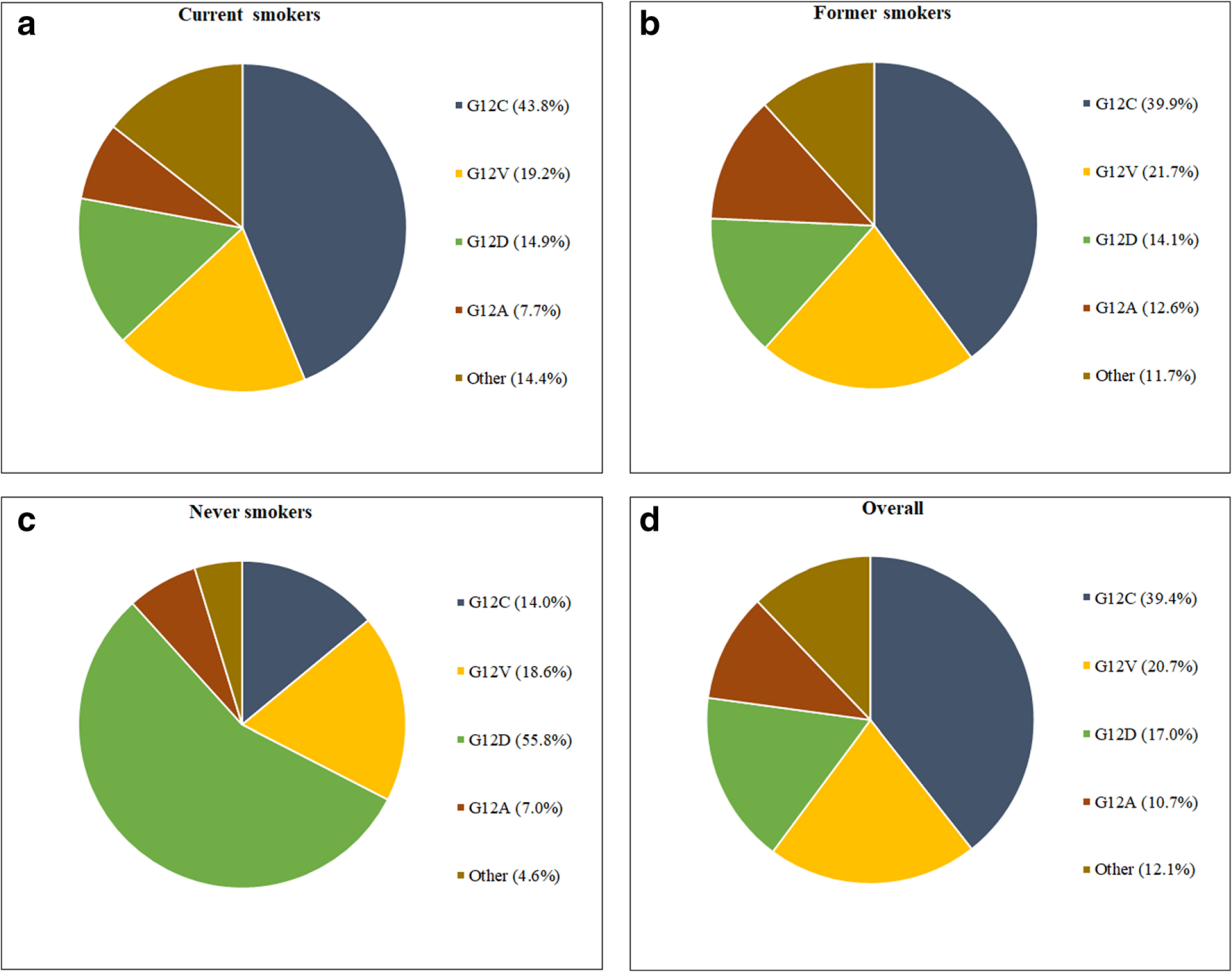
KRAS mutational subtypes and smoking history in lung adenocarcinoma (LADC) [12]. In current (**a**) and former (**b**) smokers, KRAS G12C is the most common mutation, while KRAS G12D is the most frequent mutation among never smokers (**c**). Overall (**d**), the most frequently diagnosed KRAS mutational subtype in LADC patients is KRAS G12C, followed by KRAS G12V, KRAS G12D, and KRAS G12A

Molecular profiling of LADC patients shows that specific demographic and clinicopathological characteristics are associated with the presence of KRAS mutations. As national surveys indicate, KRAS mutations mostly occur in Caucasian or African-American patients and are far less frequent in Asian patients [2, 3, 5]. Based on the findings of a pooled analysis of resected NSCLC tumors, KRAS mutations tend to be more common among women and patients of younger age, although only the latter remained significant at the multivariate analysis (*p* = 0.044) [7, 8]. Notably, however, no histology- or race-specific analyses were performed in the above study with regards to the prevalence of KRAS mutations. Interestingly, smoking also leaves a molecular fingerprint on KRAS, since transition mutations (G12D) are more frequent in never smokers, whereas transversion mutations (G12C and G12V) are more often found among former or current smokers [9, 10]. In addition, smokers tend to have genetically more complex KRAS-mutant tumors, with higher mutational burden and higher frequency of major co-occurring mutations in *TP53* or *STK11*, than those who have never smoked [10, 11]. The distribution of various KRAS mutational subtypes among patients with different smoking history is summarized in Fig. 1 [12].

Recently, attention has also been drawn to the special histology and co-occurring mutations in KRAS-mutant lung cancer. Initial studies [13, 14] reported that although in a much lower percentage, KRAS mutations might be present not only in LADC but also in squamous cell lung cancer. However, recent analysis using up-to-date differential diagnostic criteria suggests that KRAS mutations do not occur in pure pulmonary squamous cell lung carcinomas, and in case detected, it is confined to LADC components in squamous cancer [15]. The other important issue is the clinical relevance of specific KRAS mutations and the presence of these mutations in combination with others. Variations in KRAS mutation subtypes have been associated with distinct biological behaviors that can lead to different clinical outcomes [16, 17]. For example, tumors with KRAS G12C mutations exhibited higher ERK1/2 phosphorylation than those with KRAS G12D [3, 18]. In support of this, a recent study using KRAS mutation-driven mouse models demonstrated higher efficacy of the MEK inhibitor selumetinib in KRAS G12C tumors compared with KRAS G12D tumors [18]. Accordingly, distinct KRAS mutations may lead to differential induction of signal transduction cascades and thus to specific drug sensitivity profiles [19]. As for co-occurring mutations, double mutants (KRAS and EGFR/ALK/BRAF) are rare in LADC, and KRAS mutations are typically present as a single-driver mutation [20–22]. However, KRAS mutations co-occur commonly with mutations in tumor suppressor genes including *TP53*, *STK11* and *KEAP1*/*NFE2L2*, and a growing body of evidence suggests that these co-occurring mutations are associated with unique tumor characteristics and biological behaviors [23]. Taken together, the different amino acid substitutions in oncogenic KRAS and the presence of coexisting mutations highlight the need for genotype-specific analysis to identify clinically relevant subgroups of patients that may ultimately influence treatment decisions and prognosis [3].

## The prognostic nature of KRAS mutations in LADC

The prognostic power of KRAS mutation alone in the general NSCLC population remains disputed. As in other malignancies, KRAS mutation was first reported to be a negative prognostic factor in NSCLC in the 1980s [24, 25]. However, although a considerable number of publications verified this finding [26–30], these studies were heterogeneous with regards to histology, tumor stage, and methodology. Slebos [25], Ohtaki [28], and Izar [29] investigated the prognosis in completely resected LADCs, while later studies were performed in stage IIIB–IV NSCLC patients [30–32]. The strongest proof of KRAS being a negative prognostic factor in NSCLC was reported by Mascaux et al. who conducted a meta-analysis of 53 studies and found that KRAS mutation correlated with a significantly worse prognosis (hazard ratio [HR] 1.40; *p* = 0.01; HR 1.5 for LADCs; *p* = 0.02) [33]. Contrary to this, in a study analyzing 998 LADCs, 318 of which harbored KRAS mutation, the authors concluded that KRAS mutation was not an individual prognostic factor [34]. One of the most comprehensive study, a meta-analysis of four individual trials of adjuvant chemotherapy in 1500 NSCLC patients (among them 300 KRAS-mutant cases), also reported that KRAS mutation had no prognostic value in this setting [8]. However, a more recent study that involved 1935 patients reported a clear advantage in overall survival (OS) for KRAS wild type patients, although the presence of mutation did not impact progression-free survival (PFS) [30]. Another recent pooled analysis of studies assessing the role of KRAS mutation in circulating tumor DNA also indicated poorer PFS and OS in KRAS-mutated genotypes [35].

The prevalence of KRAS mutations varies among different ethnic groups and ethnicity; therefore, it might also have an impact on prognosis [5]. A meta-analysis of 41 trials and 6939 patients concluded that KRAS mutation was a negative prognostic factor in NSCLC. Not surprisingly, these authors found that KRAS mutation only had a prognostic role in LADC (HR was 1.39; 95% CI 1.24–1.55). The authors also looked at ethnicity, comparing Asians and non-Asians, and found that the HR for Asians was much larger than that for non-Asians, implying that KRAS mutations have a worse prognosis in Asian patients [36]. These results were also backed up by a recent meta-analysis including over 9000 patients [37]. Of note, patients with EGFR mutant LADC have a better prognosis, and thus KRAS mutations’ prognostic power might be influenced by the inclusion/exclusion and the proportion of EGFR mutant cases in the study cohort.

Several studies suggest that due to the heterogeneity of KRAS mutations, specific mutational subtypes might have different effects on survival and treatment response. For example, in a mutation subtype-specific analysis of 505 stage III–IV LADC patients treated with chemotherapy, our group could not demonstrate prognostic or predictive potential of KRAS mutation. However, we found that G12V mutant patients had higher response rates and slightly longer PFS [31]. On the other hand, in two retrospective studies, the authors found a significantly shorter OS in patients with KRAS G12C mutation [38]. Garassino et al. further highlighted the role of subtype-specific KRAS mutation analysis when they conducted a preclinical study assessing the *in vitro* chemosensitivity of NSCLC cells. They found that G12V mutant tumor cells were more sensitive to cisplatin and, furthermore, that G12D mutation led to increased resistance to paclitaxel and sensitivity to sorafenib, while G12C mutation was associated with reduced response to cisplatin and increased sensitivity to paclitaxel and pemetrexed [19]. Villaruz et al. found a slightly increased OS in patients with G12C mutant tumors when compared with those with tumors harboring other KRAS mutation subtypes [34]. Table 1 summarizes a selection of the major studies about the prognostic relevance of KRAS status in early- and advanced-stage NSCLC.

**Table 1:** Selected major studies about the prognostic relevance of KRAS status in lung cancer

Studies	Results (KRAS as a prognostic factor)	Pts	Treatment	Study format
Slebos et al. 1990 [25]	Negative prognostic factor	Stage I–IIIA LADC *n* = 69	Surgery	Single-center, case series
	RFS *p* = 0.038			
Mascaux et al. 2005 [33]	Negative prognostic factor	NSCLC *n* = 5216	Various	Meta-analysis (53 studies)
	OS (HR 1.5 for LADC)			
Ihle et al. 2012 [32]	Not significant	Stage IV NSCLC *n* = 215	CHT + EGFR TKI	Data from the phase II study, BATTLE trial
	G12V + G12C (*p* = 0.046) are negative prognostic factors			
Shepherd et al. 2013 [8]	Not significant	Stage I–III NSCLC *n* = 1543	Surgery/adjuvant CHT	Meta-analysis (4 studies)
	HR 1.04 G12x			
	HR 1.01 G13x			
Guan et al. 2013 [30]	Negative prognostic factor for OS but not for PFS	NSCLC *n* = 273	Surgery/CHT/CHT-RT/EGFR TKI	Single-center, retrospective, case matching
	OS (HR 2.69; *p* < 0.001), PFS (*p* = 0.27)			
Villaruz et al. 2013 [34]	Not significant	Stage I–III LADC *n* = 988	Various	Single-center, retrospective
	OS (*p* = 0.612)			
	PFS (*p* = 0.89)			
Meng et al. 2013 [36]	Negative prognostic factor	NSCLC *n* = 6939	Various	Meta-analysis (41 studies)
	HR 1.45 (95% CI 1.29–1.62)			
	Especially for early stage and Asian ethnicity			
Cserepes et al. 2014 [31]	Not significant	Stage IIIB–IV LADC *n* = 505	CHT	Single-center, retrospective
	OS (*p* = 0.917)			
	PFS (*p* = 0.534)			
Izar et al. 2014 [29]	Negative prognostic factor	Stage I LADC *n* = 312	Surgery	Single-center, retrospective
	OS (*p* = 0.0001) and DFS (*p* < 0.0001)			
Ohtaki et al. 2014 [28]	Negative prognostic factor	Stage I–IV LADC *n* = 58	Surgery	Single-center, case series
	2-year survival (18% KRAS vs. 81% EGFR vs. 47% wt)			
Renaud et al. 2016 [16]	Not significant	Stage I–IIIA NSCLC *n* = 841	Surgery/adjuvant CHT	Single-center, retrospective
	Only in G12V (OS 26 vs. 60 months; PFS 15 vs. 24 months)			
Fan at al. 2017 [35]	Negative prognostic factor	NSCLC *n* = 2293	EGFR TKI	Meta-analysis (13 studies) circulating tumor DNA
	PFS (HR = 1.83, 95% CI 1.40–2.40, *p* < 0.0001) and OS (HR = 2.07, 95% CI 1.54–2.78, *p* < 0.00001)			

*LADC*, lung adenocarcinoma; *CHT*, chemotherapy; *CHT-RT*, chemotherapy and radiation therapy; *EGFR*, epidermal growth factor receptor; *HR*, hazard ratio; *KRAS*, Kirsten rat sarcoma viral oncogene homolog; *mut*, mutant; *NSCLC*, non-small cell lung cancer; *PFS*, progression-free survival; *pt*, patient; *RFS*, recurrence-free survival; *TKI*, tyrosine kinase inhibitor; *wt*, wild type

## Predictive role of KRAS mutations

### Predictive value of KRAS mutations for response to chemotherapy

Despite the recent developments in NSCLC therapy, most patients with advanced-stage disease still receive platinum-based chemotherapy. Most studies do not suggest KRAS mutation as a predictive biomarker for response to chemotherapy. The predictive value of KRAS mutation in NSCLC was investigated in the metastatic setting in patients receiving definitive chemotherapy [39], in patients receiving adjuvant chemotherapy with radiation after surgery [40], and also in the phase III TRIBUTE trial where first-line carboplatin/paclitaxel plus erlotinib or placebo was compared in advanced-stage NSCLC [41]. In none of the above settings did KRAS prove to be a predictive factor for response rate, PFS, or OS.

More recently, results of the JBR10 trial, which studied the effects of postoperative vinorelbine or cisplatin in patients with resected stage IB or II NSCLC, were published. Remarkable benefit from chemotherapy was only reported in KRAS wild type patients; however, the difference did not prove to be statistically significant (*p* = 0.29) [42]. Neoadjuvant and perioperative chemotherapy sequences with carboplatin/paclitaxel or cisplatin/gemcitabine were compared in the phase III IFCT-0002 trial. KRAS-mutant tumors were shown to exhibit lower response to cytotoxic chemotherapy in univariate analysis, although KRAS mutation was not a significant predictor in multivariate analysis [43]. A recent retrospective analysis of patients with advanced-stage NSCLC also found that KRAS mutation is a predictor for poor OS when treated with cytotoxic chemotherapy [44]. Furthermore, it was shown that the co-existence of *TP53* mutation predicts worse outcome [45]. Another aspect was shown in a study conducted in an Asian cohort that analyzed outcomes in patients receiving different chemotherapeutic regimens according to the KRAS mutation status. Significantly poorer PFSs and OSs were seen in patients with KRAS mutations when treated with pemetrexed or gemcitabine but not in those receiving taxanes [46]. Of note, a potential negative effect of KRAS codon 13 mutations was suggested by a clinical study [8] showing significantly shorter PFS and OS in patients with such mutations. As previously mentioned, in a preclinical study by Garassino et al., similar results were seen [19].

In summary, although KRAS mutations can be potentially considered as predictive biomarkers for chemotherapy in LADC, the exact type of mutation and the type of chemotherapy should also be taken into consideration.

### Predictive value of KRAS mutations for response to targeted therapy

One of the major debates over the predictive role of KRAS-mutant status of NSCLC patients takes place in the field of EGFR-targeted therapies [5]. Most published data, including a meta-analysis of 22 studies, suggest that KRAS mutational status is a significant negative predictor for EGFR tyrosine kinase inhibitors (TKIs) [41, 47–49]. Accordingly, KRAS-mutated patients treated with EGFR TKIs have a trend for worse objective response rates (ORR), PFS, and OS compared with patients without KRAS mutation [41, 48, 49]. However, despite the convincing results, controversies still exist and not all studies have reached the same conclusions [46, 50]. A possible explanation for these discrepancies in the literature might be that the response to EGFR TKI is greatly influenced not only by the presence or absence of KRAS mutations but also by the involved KRAS codons and the type of amino acid substitutions [5, 51]. In support of this, a recent study showed poorer treatment efficacy in the case of G12C and G12V KRAS mutations but promising response rates in G12D and G12S KRAS-mutant NSCLC patients treated with EGFR TKIs [52]. All in all, patients with KRAS-mutant NSCLC generally have a poor response to EGFR inhibitors; however, due to the heterogeneity of various KRAS mutations, KRAS mutational analysis cannot be recommended as a tool to select NSCLC patients for EGFR TKI therapy.

### Predictive value of KRAS mutations for response to anti-vascular therapy

Although the RAS pathway has been shown to affect VEGF expression, very few studies investigated the influence of KRAS mutation on the efficacy of anti-angiogenic therapy [53]. Only two groups reported that G12V, G12A [54], and G12D [55] KRAS mutations are associated with poor outcome in patients with colorectal cancer (CRC) receiving bevacizumab. As for NSCLC, a phase II trial evaluated the addition of neoadjuvant bevacizumab to chemotherapy and found that no patients with KRAS mutation (0 out of 10) demonstrated pathological response to neoadjuvant bevacizumab and chemotherapy, while 35% of KRAS wild type patients had significant response [56]. Furthermore, in a recent single-center retrospective study from our group, KRAS mutation, and especially G12D mutation, was shown to be a predictor of significantly worse PFS and OS in advanced-stage NSCLC patients treated with bevacizumab plus platinum-based chemotherapy [57]. Table 2 summarizes the available data on the predictive value of KRAS mutations for therapeutic response in NSCLC.

**Table 2:** Selected major studies about predictive role of KRAS mutations in lung cancer

Study	Pts tested for KRAS	KRAS status		Treatment	Endpoint	KRAS status	
		KRAS mut	KRAS WT			KRAS mut	KRAS WT
Rodenhius et al. 1997 [39]^#^	62 (stage III–IV)	16	46	Carboplatin + ifosfamide + etoposide	ORR%	19	26
					PFS*	4	5
					OS*	8	9
Schiller et al. 2001 [40]	184 (stage II–IIIA)	44	140	Cisplatin + etoposide	OS	24.7	42
Eberhard et al. 2005 [41]	133 (advanced stage)	25	108	Carboplatin + paclitaxel + erlotinib	ORR%	23	26
					PFS	6	5.4
					OS	13.5	11.3
Khambata-Ford et al. 2010 [122]	202 (stage IIIB–IV)	35	167	Taxane + carboplatin + cetuximab	ORR%	30.80	32.90
					PFS	5.60	5.10
					OS	16.8	9.7
Ludovini et al. 2011 [123]	166 (stage III–IV)	11	151	EGFR TKI	ORR%	0	35.7
					PFS	2.7	5.6
					OS	19.3	28.6
Fiala et al. 2013 [124]	448 (stage IIIB–IV)	69 (G12C: 38)	379	EGFR TKI	PFS (weeks)	4.3 (G12C) vs. 9.0 (non-G12C)	
					OS (weeks)	12.1 (G12C) vs. 9.3 (non-G12C)	
Zer et al. 2016 [52]	785 (stage IIIB–IV)	155	630	EGFR TKI (pooled analysis)	OS	4.5	6
Hames et al. 2016 [44]	150 (stage IV)	80	70	Standard CHT	PFS	4.7	5.7
					OS	8.8	13.5
Dong et al. 2017 [59]^#^	34 (not specified)	8	26	Pembrolizumab	ORR%	25	6.6
	20 (not specified)	5	15	Pembrolizumab or nivolumab	PFS	14.7	3.5
Gettinger et al. 2018 [63]	129 (advanced stage)	8	13	Nivolumab	5-year survival	18%	25%
Ghimessy et al. 2019 [57]^#^	247 (stage IIIB–IV)	95	152	Standard CHT + bevacizumab	PFS	7.03	8.63
					OS	14.23	21.57

*CHT*, chemotherapy; *EGFR*, epidermal growth factor receptor; *KRAS*, Kirsten rat sarcoma viral oncogene homolog; *mut*, mutant; *PFS*, progression-free survival; *pt*, patient; *TKI*, tyrosine kinase inhibitor; *wt*, wild type

*In months (unless otherwise stated)

^#^Study with LADC only

### Predictive value of KRAS mutations for response to immune checkpoint inhibition therapy

Programmed cell death protein 1 (PD-1) expression has been shown to be in close connection with KRAS status, and KRAS mutations were described as possible biomarkers for immune checkpoint inhibitors [58]. Also, a clinical benefit was reported to PD-1 inhibitors in KRAS-mutant patients [59]. The elevated expression of programmed cell death ligand 1 (PD-L1) has been demonstrated in KRAS-mutant cells, and it was also shown that ERK activation mediates the upregulation of PD-L1 by KRAS mutations [60]. On the contrary, Reiniger et al. did not find significant relations between PD-L1 expression and KRAS status in LADC [61]. It was also reported that pembrolizumab (a PD-1 inhibitor) or an ERK inhibitor might prevent CD3+ T cells becoming apoptotic by recovering tumor immunity thus preventing immune escape [62]. In another study, however, Gettinger et al. found increased response and survival with nivolumab monotherapy and driver mutations of EGFR or KRAS did not show significant effect on survival or treatment response [63]. Altogether, further clinical experience is needed to determine whether KRAS mutation is a useful predictive factor for immunotherapy in NSCLC. Table 2 includes three trials where the predictive role of the KRAS mutational status for immune checkpoint inhibition therapy was studied.

## KRAS as a therapeutic target in NSCLC

### Pitfalls of KRAS mutation targeting in NSCLC

**Fig. 2:**
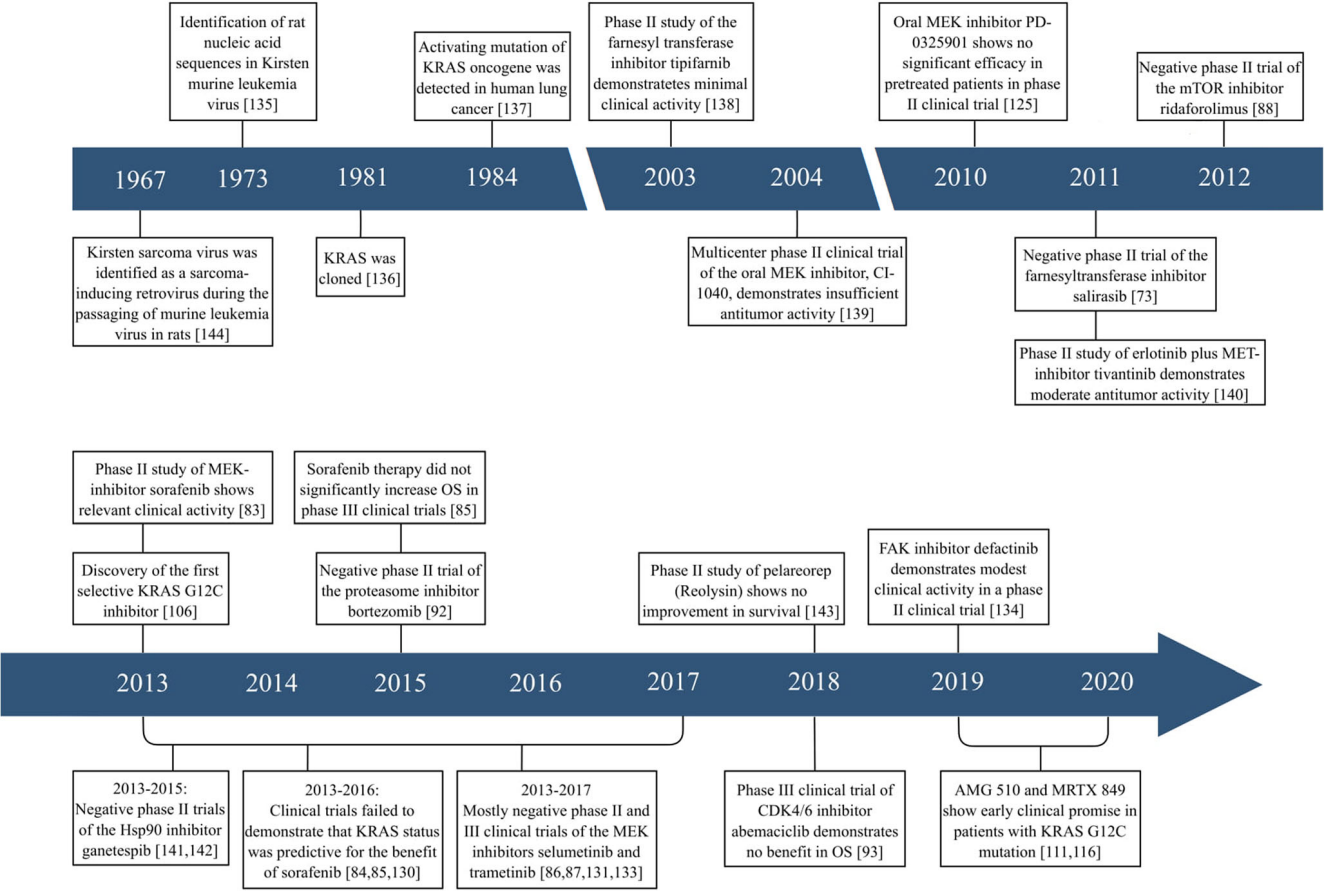
A chronicle of KRAS mutation in lung cancer. Major biological discoveries and key clinical trials. During its more than 30-year history, our knowledge of KRAS mutation in lung cancer has progressed through a series of phases. Although the relationship between RAS genes and lung cancer was described in 1984, the first clinical trials investigating the efficacy of indirect KRAS inhibitors were carried out only in the early 2000s. Since then, large numbers of both direct and indirect KRAS inhibitors have been developed and tested. However, until recently, efforts to target the RAS family proteins were mostly ineffective in the clinics. At the same time, in the past years, a worldwide awakening of interest led to rapid translational progress and to the discovery of novel direct covalent KRAS G12C-inhibitors, some of which have been tested in clinical trials. The renewed enthusiasm and biological and clinical progress have changed the landscape of KRAS-mutated lung cancer and have led to the first serious discussions of whether RAS is indeed a druggable target. KRAS, Kirsten rat sarcoma viral oncogene homolog; MEK, MAPK/ERK kinase; mTOR, mammalian target of rapamycin; MET, MET proto-oncogene; Hsp90, heat shock protein 90; CDK4/6, cyclin-dependent kinases 4/6; FAK, focal adhesion kinase; OS, overall survival

Because of its high mutation frequency in NSCLC, KRAS is an appealing target. However, the development of targeted therapies for KRAS-mutant lung cancers has long been marked by frustration [64–66]. For decades, KRAS was considered undruggable due to its exceptionally high affinity to GTP/GDP, to the absence of known allosteric binding sites, and to the presence of extensive post-transcriptional modifications [3, 7, 67]. KRAS protein shows high resistance against small-molecule modulation, since it is a small protein with a relatively smooth surface without clear binding pockets (besides its GTP/GDP binding pocket) [68]. Under physiological conditions *in vivo*, GTP almost exclusively occupies all potential binding sites with extremely high affinity. The development of KRAS inhibitors that achieve adequate blood concentration enough to displace GTP is, therefore, an almost improbable task [68, 69]. In addition, the binding of small-molecule inhibitors is also influenced by the interactions of KRAS with other proteins that make the surface of the KRAS protein shallow [68]. Importantly, indirect targeting of the molecules within the KRAS signaling pathway also proved to be almost ineffective due to the complexity and biological heterogeneity of KRAS mutations in NSCLC [68, 70]. All in all, despite enormous efforts, to date, almost all identified compounds that could effectively and directly target mutant KRAS have failed. However, with new technologies in drug development and novel mechanistic insights into RAS biology, new targeted therapeutic agents are under development with promising preclinical activity (Fig. 2).

### Targeting KRAS membrane anchorage

RAS proteins require membrane associations to become biologically active [11, 68, 71]. The membrane anchorage of KRAS is dependent on posttranslational modification of the CAAX motif by farnesyltransferases. Initial preclinical studies with farnesyltransferase inhibitors (FTIs) demonstrated moderate success in blocking tumor cells both *in vitro* and *in vivo*. However, in the presence of FTIs, KRAS can be alternatively prenylated by geranylgeranyl-transferase-I, thus overcoming the effect of farnesyltransferase inhibition [6, 11, 72]. As expected, these results foreshadowed the disappointing clinical trials with FTIs that failed to improve outcomes in KRAS-mutant LADC patients [11, 73]. Still, some novel FTIs, when combined with other inhibitors such as geranylgeranyl-transferase inhibitors, showed potent anti-cancer activities in KRAS-driven pancreatic tumors. Nevertheless, the efficacy of these dual-functional therapeutic agents has not yet been investigated in LADC [74, 75]. Preclinical studies indicated that lung cancer cells might be sensitive to prenylation inhibition by bisphosphonates [76, 77]. Additionally, oral bisphosphonate use was associated with lower lung cancer risk among never smoker postmenopausal women in a large prospective study [78]. In isolated clinical cases, bisphosphonate therapy caused the regression of the primary lesion and its hepatic metastases in LADC [79]. Notably, however, a recent preclinical study demonstrated that the aminobisphosphonate compound zoledronic acid was ineffective in NSCLC cells harboring exon 2 codon 12 KRAS mutation, since this mutational subtype leads to prenylation-independent activation of KRAS. [80]. Nevertheless, the impact of the bisphosphonate treatment in KRAS-mutant LADC patients remains to be fully explored. Similarly, targeting other enzymes involved in the post-prenylation processing of RAS (e.g., the RAS converting CAAX endopeptidase 1 [Rce1] and isoprenylcysteine carboxyl methyltransferase [ICMT]) could as well inhibit the RAS-driven tumorigenesis [81]. In the past years, numerous Rce1 and ICMT inhibitors have been designed and investigated in several RAS-driven tumor entities. However, despite the encouraging results *in vitro*, the use of Rce1 and ICMT inhibitors could impact the normal function of other proteins as well *in vivo*, raising the questions about normal tissue toxicity and possible side effects of these inhibitors [82].

### Targeting KRAS downstream signaling pathways

Another feasible approach to treat KRAS-mutated NSCLC might be to target the main signaling pathways controlled by the constitutively active mutant KRAS (i.e., the RAF-MEK-ERK or the PI3K/AKT/mTOR pathways). The inhibitors of these signaling pathways have been tested in different RAS-driven tumor types, and some of them showed promising activity in preclinical models [11]. The results of conducted trials in KRAS-mutant NSCLC in regards to downstream signaling pathway inhibition are summarized in Table 3. Notably, one of the most promising therapeutic agent was sorafenib, a multikinase inhibitor that showed promising results in preclinical settings and phase II clinical trials but only modest clinical activity in phase III trials with ORRs generally less than 10% and median PFS of approximately 3 months [11, 83–85]. Clinical outcomes for single-agent allosteric MEK inhibitors were also discouraging, since no clinical activity of selumetinib or trametinib was observed [86, 87]. As for other downstream inhibitors, the mTOR inhibitor ridaforolimus showed a moderate increase in PFS, but its clinical benefit was questionable with several side effects [88]. All in all, clinical trials investigating the efficacy of KRAS downstream inhibitors in monotherapy provided limited clinical benefit and substantial toxicity in most studies [11, 65] Yet, recent preclinical studies with patient-derived xenograft tumors highlighted the need for combination therapy in order to fully block KRAS signaling in lung cancer [89]. These results provide a strong therapeutic rationale to treat epithelial KRAS-mutant lung cancer with ERBB and MEK inhibitors, and mesenchymal-like KRAS-mutant lung cancer by combined therapy with FGFR and MEK inhibitors [3, 89]. To date, however, none of these findings have been translated into the clinics.

**Table 3:** Completed clinical trials evaluating the efficacy of therapeutic agents targeting the downstream effectors of the KRAS pathway in lung cancer

Therapeutic agent	Date	Study design	Target	Pts*	KRAS status	Primary endpoint	Median PFS (months)^#^	Median OS (months)^#^
PD-0325901 Haura et al. [125]	2010	Phase II, OL, MC	MEK	34	NA	Response	1.8	7.8
Sorafenib Smit et al. [126]	2010	Phase I, OL, SC	RAS/RAF	10	mut	Response	3	NA
Selumetinib Hainsworth et al. [127]	2010	Phase II, R, OL, MC	MEK	84	NA	DPE	67 vs. 90 days; *p* = 0.79	NA
Ridaforolimus Riely et al. [88]	2012	Phase II, R, OL, MC	mTOR	79	mut	PFS	4 vs. 2; *p* = 0.013	18 vs. 5; *p* = NS
RO5126766 Martinez-Garcia et al. [128]	2012	Phase I, OL, MC	MEK/RAF	3	NA	Safety	NA	NA
Sorafenib Dingemans et al. [83]	2012	Phase II, OL, MC	RAS/RAF	59	mut	DCR at 6 wks	2.3	5.3
RO5126766 Honda et al. [129]	2013	Phase I, OL, SC	MEK/RAF	3	NA	Safety	NA	NA
Sorafenib Blumenschein et al. [130]	2013	Phase II, OL, MC	RAS/RAF	105	mut, NA	OS	2.83	8.48
Selumetinib Jänne et al. [131]	2013	Phase II, R, MC	MEK	87	mut	OS	5.3 vs. 2.1; *p* = 0.014	9.4 vs. 5.2; *p* = NS
Sorafenib Paz-Ares et al. [85]	2015	Phase III, R, DB, MC	RAS/RAF	703	mut, wt	OS	KRAS mut pts. 2.6 vs. 1.7; *p* = 0.007	KRAS mut pts. 6.4 vs. 5.1; *p* = NS
Trametinib Blumenschein et al. [87]	2015	Phase II, R, OL, MC	MEK	129	mut	PFS	12 wks. vs. 11 wks.; *p* = NS	8 wks. vs. not reached; *p* = NS
Sorafenib Papadimitrakopoulou et al. [84]	2016	Phase II, R, OL, MC	RAS/RAF	200	mut, wt	DCR at 8 weeks	NS between KRAS mut and KRAS wt pts.	NS between KRAS mut and KRAS wt pts.
Selumetinib Carter et al. [86]	2016	Phase II, R, OL, MC	MEK	89	mut, wt	PFS, Response	KRAS mut pts. 4 vs. 2.3; *p* = NS	KRAS mut pts. 10.5 vs. 21.8; *p* = NS
RO5126766 Chenard-Poirier et al. [132]	2017	Phase I, OL, SC	MEK/RAF	10	mut	Response	NA	NA
Selumetinib Jänne et al. [133]	2017	Phase III, R, DB, MC	MEK	510	mut	PFS	3.9 vs. 2.8; *p* = NS	8.7 vs. 7.9; *p* = NS
Defactinib Gerber et al. [134]	2019	Phase II, OL, SC	FAK	55	mut	PFS at 12 wks	45 days	NA

*DB*, double blind; *DCR*, disease control rate; *DPE*, disease progression event count; *FAK*, focal adhesion kinase; *KRAS*, Kirsten rat sarcoma viral oncogene homolog; *MC*, multicenter; *MEK*, MAPK/ERK kinase; *mTOR*, mammalian target of rapamycin; *mut*, mutant; *NS*, non-significant; *NA*, not announced; *OL*, open label; *OS*, overall survival; *PFS*, progression-free survival; *pt*, patient; *R*, randomized; *RAF*, rapidly accelerated fibrosarcoma; *RAS*, rat sarcoma virus; *SC*, single-center; *wks*, weeks; *wt*, wild type

*Patients with non-small cell lung cancer

#In months unless otherwise stated

### Synthetic lethal vulnerabilities in KRAS-mutant NSCLC

An alternative approach to direct targeting of KRAS-mutant cancer genes involves targeting co-dependent vulnerabilities or synthetic lethal partners that are preferentially essential for KRAS oncogenesis [90]. The therapeutic ablation of these secondary targets would hypothetically result in the selective death of KRAS-mutant but not KRAS wild type tumor cells [11, 91]. One of the therapeutic approaches inducing synthetic lethality included the proteasome inhibitor bortezomib [7]. However, in a small phase II clinical trial of 16 NSCLC patients with KRAS G12D mutation, bortezomib showed only a modest disease control rate of 40%, only 1 objective response (ORR 6%), and a PFS of 1 month [92]. The pharmacological inhibition of cyclin-dependent kinase (CDK) was as well of great clinical interest in the past years, and the selective CDK4/6 inhibitor abemaciclib showed indeed promising results both in phases I and III clinical trials. Accordingly, abemaciclib demonstrated significantly higher ORRs and PFS than erlotinib in pretreated patients with advanced-stage KRAS-mutant lung cancer patients, but no significant difference was observed in OS [93, 94]. The efficacy of other CDK4/6 inhibitors (including palbociclib in combination therapy with MEK inhibitors) is currently under investigation (NCT02022982 and NCT03170206). Finally, preclinical studies suggest that dual inhibition of discoidin domain receptor 1 (DDR1) and Notch pathways also hampers the growth of murine and human KRAS-mutant LADC; however, these results have not been yet validated in clinical trials [95].

### Targeting direct regulators of KRAS activity

RAS protein is transformed into its active, GTP-bound state by interaction with guanine nucleotide exchange factors (GEFs) [96, 97]. The most-studied GEF for RAS is the protein Son of Sevenless (SOS) (for which two isoforms, SOS1 and SOS2, are known), which catalyzes the release of GDP and allows the binding of the more abundant GTP [96, 97]. Accordingly, the selective inhibition of SOS1 with small-molecule inhibitors such as the experimental BI 1701963 might allow KRAS blockade irrespective of KRAS mutation type [96, 98–100]. This highly specific SOS1 inhibitor reduces both KRAS-GTP levels and MAPK signaling in cellular and animal models [100, 101]. Furthermore, preclinical studies also suggest that BI 1701963 indeed blocks tumor growth both in G12 and G13 KRAS-mutant tumors, and the compound is selective for KRAS-mutant cell lines [100, 101]. The efficacy of the SOS1-binding pan-KRAS inhibitor BI 1701963 alone or in combination with the MEK inhibitor trametinib in patients with KRAS-mutated solid tumors is currently under investigation (NCT04111458).

### Direct targeting of mutant KRAS

KRAS has been historically acknowledged a non-druggable target. However, according to the results of the latest preclinical findings, the landscape of G12C KRAS-mutated lung cancer might change. After the discovery of new allosteric regulatory pockets in GDP-RAS adjacent to the cysteine residue of KRAS G12C, compounds that target the guanine nucleotide-binding pocket (SML-8-73-1) or allele-specific inhibitors (ARS-853) have been reported [102–104]. Of note, the effects of both SML-8-73-1 and ARS-853 on mutant KRAS G12C are irreversible. SML-8-73-1 can covalently react with KRAS G12C, thus competing with GTP and GDP for active site binding in a cellular context even in the presence of a very high concentration of GTP [105]. Accordingly, by locking the KRAS-GDP state, these GDP-derived inhibitors can block the proliferative activity of the KRAS-mutant cells [103, 104]. Despite their preclinical inhibitory effects on KRAS G12C, follow-up studies also showed that the specificity of these inhibitors is somewhat low and may have off-target effects when used in the clinics [103–105]. ARS-853, on the other hand, does not compete with GTP for binding to KRAS, since it binds to a pocket nearby the nucleotide-binding pocket [106]. Hence, by making KRAS more preferential to accept GDP binding rather than GTP, it reduces the KRAS-GTP levels by more than 90% and increases the *in vitro* hydrolytic reaction and thus locking the KRAS in the GDP-bound state [103, 104, 106]. Accordingly, ARS-853 inactivates the RAS signaling by a trapping mechanism, by which KRAS G12C is trapped in the KRAS-GDP state [103, 104]. Importantly, similarly to SML-8-73-1 and SML-10-70-1, ARS-853 only binds to KRAS G12C and has no inhibitory effects on wild type KRAS and other types of mutant KRAS [68]. These findings were recently translated into mouse model studies where ARS-1620, a similar covalent compound with high potency and selectivity for KRAS G12C, induced durable tumor regression in different patient-derived tumor models [107]. Furthermore, recent studies also suggest a potential synergistic activity when ARS-853 is combined with receptor TKIs such as EGFR TKIs, indicating that covalent G12C-specific inhibitors might indeed be promising therapeutic agents used for the treatment of KRAS G12C-mutant NSCLC patients [103, 104, 108, 109]. To date, however, no clinical trials have been communicated with ARS-853 or ARS-1620 in KRAS-mutant NSCLC.

### Novel direct covalent KRAS-G12C inhibitors: promising preclinical and clinical results

Recent discoveries of the aforementioned covalent KRAS G12C-specific inhibitors have led to the first serious discussions of whether RAS is indeed a druggable target. AMG 510 is a novel small molecule that covalently binds to the cysteine amino acid of KRAS G12C-mutant proteins, and thus, it locks KRAS in its inactive GDP-bound state irreversibly [110, 111]. In preclinical studies, treatment with AMG 510 induced the regression of KRAS G12C tumors and improved the efficacy of both chemotherapy and targeted agents [112]. Furthermore, AMG 510 therapy also resulted in a pro-inflammatory tumor microenvironment in immune-competent mice and produced durable responses alone and in combination with immune checkpoint inhibitors as well [112, 113]. As for its clinical benefit, in a recent phase I clinical trial in a small number of pretreated NSCLC patients (NCT03600883), a partial response was achieved in 54% and stable disease in 46% of the patients with a disease control rate of 100% [66, 111]. Importantly, the treatment was well-tolerated with the absence of dose-limiting toxicity and the occurrence of only a few drug-related side effects [66, 111]. A multicenter phase II clinical trial is currently ongoing [66]. MRTX 849 is another potent, mutation-selective, and orally available irreversible small-molecule inhibitor of KRAS G12C [114]. MRTX 894 also locks KRAS in an inactive GDP-bound state and blocks the KRAS-dependent signal transduction and cancer cell viability [3, 68]. In preclinical *in vivo* models, MRTX 894 treatment was associated with potent antitumor activity in different KRAS G12C-positive patient- and cell-derived tumors, with an overall response rate of 65% [114–116]. Meanwhile, with regards to its clinical efficacy, the first results of an ongoing phase I/II clinical trial (NCT 03785249) suggest promising clinical outcomes (especially in NSCLC patients) and favorable safety profile [68, 116]. Another potential direct KRAS G12C inhibitor might be the investigational, orally available JNJ-74699157 (ARS-3248), which is a new generation of the KRAS G12C inhibitor ARS-1620. A multicenter phase I clinical trial (NCT04006301) evaluating JNJ-74699157 started the enrollment in July 2019 and is currently ongoing [66, 68]. Further potential KRAS G12C inhibitors under development include the Eli Lilly drug LY3499446 (NCT04165031), the Pfizer drug tetrahydroquinazoline derivatives (US 2019/0248767A1), and the AstraZeneca drug tetracyclic compounds (WO 2019/110751 A1) [68].

### Other therapeutic approaches to treat KRAS-mutant NSCLC

Besides the need to develop new, single-agent therapeutic compounds, the complexity of the RAS signaling pathway underscores the necessity for a variety of combination therapy as well. Consequently, combination screenings have been conducted using ARS-1620, AMG 510, and MRTX 849 to identify combinations that may enhance the therapeutic response [68, 109]. Accordingly, adding mTOR and IGF1R inhibitors to ARS-1620 greatly improves its effectiveness on KRAS G12C-mutant lung cancer cells *in vitro* and in mouse models [109]. Meanwhile, the combination of AMG 510 with multiple agents including different MEK inhibitors or the standard of care chemotherapeutic agent carboplatin resulted in the synergistic killing of tumor cells *in vitro*, thus providing rationale for this approach in the clinic [112]. As for the later-mentioned direct KRAS G12C inhibitor, combinations of MRTX 849 with agents including the HER family inhibitor afatinib, the CDK4/6 inhibitor palbociclib, the SHP2 inhibitor RMC-4550, and different mTOR pathway inhibitors demonstrated enhanced response and marked tumor regression in several cell-line panels and tumor models, including MRTX 849-refractory models as well [115]. Finally, since preclinical works support the hypothesis that KRAS mutations may be vulnerable to immune checkpoint inhibition, the evaluation of clinical response to combination therapy of direct and indirect KRAS inhibitors and immune checkpoint inhibitors is also justified [117].

As for other therapeutic agents, AZD4785 is a KRAS antisense oligonucleotide that targets the KRAS gene irrespective of its mutational status, thereby inhibiting the downstream effector pathways [118]. Despite the encouraging preclinical results showing significant antitumor activity and favorable safety profile in mice and monkeys bearing KRAS-mutant lung cancer, the first phase I clinical trial (NCT03101839) failed, possibly because AZD4785 targets both mutant and wild type KRAS protein [3, 118]. Accordingly, the development of AZD4785 was later discontinued. As RAS proteins are highly immunogenic, another potential therapeutic approach might be the adoptive transfer of genetically engineered tumor antigen-specific T cells into patients with KRAS-mutant tumors [119]. Pharmacological studies are still in a very early stage; however, the first results indeed show an *in vitro* efficacy of G12V-reactive CD4+ T cells against KRAS G12V-mutant NSCLC cells [66, 120].

## Open questions and future challenges

While direct KRAS G12C inhibitors have shown promising results in some solid tumors including LADC, the development of new potential therapeutic strategies for the treatment of KRAS-mutated lung cancer is a work in progress, and many questions remain.

1. Despite the promising results achieved with direct KRAS G12C inhibitors, approximately half of the G12C-mutant lung cancer patients show only a partial response to these therapeutic agents [121]. The mechanism of how cancer cells bypass inhibition to prevent maximal response to therapy is not yet fully understood. A possible explanation might be that some quiescent cells produce new KRAS G12C in response to suppressed mitogen-activated protein kinase output, which is maintained in its active, drug-insensitive state by the epidermal growth factor receptor and aurora kinase signaling [121]. Since the inhibitors bind only to the inactive conformation of KRAS, the cells with these adaptive changes bypass the effects of KRAS G12C inhibitors and resume to proliferate [121]. This adaptive process must be overcome if we are to achieve complete and durable responses in the clinic [121].

2. Distant organ metastases with unique microenvironmental features occur frequently in lung cancer. Some of these special microenvironments, including the blood-brain barrier in case of brain metastases, represent a potential challenge for targeted therapeutic agents to reach the tumor cells in appropriate concentrations. Thereby, it is still an open question which of the direct covalent KRAS inhibitors will be able to penetrate the blood-brain barrier or other metastatic site-specific barriers.

3. Whether direct inhibition of KRAS with these new compounds in monotherapy is sufficient also remains an open question. Accordingly, future studies evaluating the clinical efficacy and tolerability of direct covalent KRAS inhibitors in combination therapy with anti-EGFR therapies, immune checkpoint inhibitors, or upstream and downstream RAS signaling inhibitors are needed.

4. Although KRAS G12C inhibitors are putting KRAS’s non-druggability reputation to the test, only 35 to 45% of all KRAS-mutant LADC patients harbor this variant [12]. Therefore, selective inhibitors or broader-acting pan-KRAS agents are needed for patients with non-G12C KRAS mutations. In non-clinical studies, the novel SOS1 inhibitors demonstrated increased antitumor activity irrespective of KRAS mutation type, yet these findings were not yet translated into the clinics.

## Conclusions

To summarize, although KRAS mutations represent one of the most common oncogenic driver mutations in lung cancer, KRAS has been historically acknowledged a non-druggable target. Indeed, to date, no effective RAS inhibitors are used in routine clinical practice. Furthermore, the predictive role of KRAS mutation in patients receiving chemo-, targeted, anti-vascular, or immunotherapy needs to be clarified. Nevertheless, recent data on the novel direct covalent KRAS G12C inhibitors AMG 510 and MRTX 849 appear to be promising both in preclinical and clinical settings. Other therapeutic approaches such as combinatory therapy with targeted agents, immune checkpoint inhibitors, KRAS downstream inhibitors, or the newly developed direct covalent inhibitors are also encouraging but require further clinical testing. At the same time, mechanisms of adaptive resistance that limits the therapeutic potential of conformation-specific KRAS G12C inhibition might represent a possible future challenge that must be overcome for durable responses. All in all, despite the historical lack of progress, the emergence of new promising agents might change the therapeutic landscape of KRAS-mutant LADC. Yet, many questions remain and the clinical relevance of KRAS gene mutations warrants further investigations.

## Data Availability

Not applicable.
